# Clinical Evaluation of the Cepheid Xpert TV Assay for Detection of Trichomonas vaginalis with Prospectively Collected Specimens from Men and Women

**DOI:** 10.1128/JCM.01091-17

**Published:** 2018-01-24

**Authors:** Jane R. Schwebke, C. A. Gaydos, T. Davis, J. Marrazzo, D. Furgerson, S. N. Taylor, B. Smith, L. H. Bachmann, R. Ackerman, T. Spurrell, D. Ferris, C.-A. D. Burnham, H. Reno, J. Lebed, D. Eisenberg, P. Kerndt, S. Philip, J. Jordan, N. Quigley

**Affiliations:** a Department of Medicine, University of Alabama at Birmingham, Birmingham, Alabama, USA; b Johns Hopkins University, Baltimore, Maryland, USA; c Indiana University School of Medicine, Indianapolis, Indiana, USA; d Planned Parenthood Mar Monte, San Jose, California, USA; e Louisiana State University Health Sciences Center, New Orleans, Louisiana, USA; f Planned Parenthood Gulf Coast, Houston, Texas, USA; g Wake Forest University Health Sciences, Winston-Salem, North Carolina, USA; h Comprehensive Clinical Trials, W. Palm Beach, Florida, USA; i Planned Parenthood of Southern New England, New Haven, Connecticut, USA; j Augusta University, Augusta, Georgia, USA; k Washington University in St. Louis, St. Louis, Missouri, USA; l Planned Parenthood Southeastern PA, Philadelphia, Pennsylvania, USA; m Planned Parenthood St. Louis Region, St. Louis, Missouri, USA; n University of Southern California, Los Angeles, California, USA; o San Francisco Public Health, San Francisco, California, USA; p George Washington University School of Public Health, Washington, DC, USA; q Geneuity, Maryville, Tennessee, USA; University of Texas Medical Branch

**Keywords:** NAAT, diagnosis, female, male, trichomonas

## Abstract

Trichomoniasis is the most prevalent curable sexually transmitted disease (STD). It has been associated with preterm birth and the acquisition and transmission of HIV. Recently, nucleic acid amplification tests (NAAT) have been FDA cleared in the United States for detection of Trichomonas vaginalis in specimens from both women and men. This study reports the results of a multicenter study recently conducted using the Xpert TV (T. vaginalis) assay to test specimens from both men and women. On-demand results were available in as little as 40 min for positive specimens. A total of 1,867 women and 4,791 men were eligible for inclusion in the analysis. In women, the performance of the Xpert TV assay was compared to the patient infected status (PIS) derived from the results of InPouch TV broth culture and Aptima NAAT for T. vaginalis. The diagnostic sensitivities and specificities of the Xpert TV assay for the combined female specimens (urine samples, self-collected vaginal swabs, and endocervical swabs) ranged from 99.5 to 100% and 99.4 to 99.9%, respectively. For male urine samples, the diagnostic sensitivity and specificity were 97.2% and 99.9%, respectively, compared to PIS results derived from the results of broth culture for T. vaginalis and bidirectional gene sequencing of amplicons. Excellent performance characteristics were seen using both female and male specimens. The ease of using the Xpert TV assay should result in opportunities for enhanced screening for T. vaginalis in both men and women and, hopefully, improved control of this infection.

## INTRODUCTION

Trichomonas vaginalis is a flagellated protozoan parasite that causes genitourinary infections in women and men. It is the most prevalent curable sexually transmitted disease (STD) and is easily treated with inexpensive antibiotics (1, 2). Trichomoniasis causes distressing symptoms like vaginal discharge and irritation in women and urethritis in men (3, 4). It is also significantly associated with an increased risk of preterm birth and the acquisition and transmission of STDs, including HIV, as well as being associated with infertility (5–8). Currently, however, there is no national control program for this infection. Until recently, the tests available for detection of the parasite were relatively insensitive (direct microscopy and antigen detection) or not widely available (culture) (9, 10). The advent and FDA clearance of nucleic acid amplification tests (NAAT) for diagnosis of T. vaginalis in women (urine [UR] samples, endocervical swab [ES] specimens, vaginal swab [VS] specimens, and patient-collected vaginal swab [PC-VS] specimens) has greatly improved our diagnostic capability, but until now there was no FDA-cleared test for detection of T. vaginalis in male urine samples (11). The GeneXpert system (Cepheid, Sunnyvale, CA) is already used to diagnose other STDs, such as Neisseria gonorrhoeae and Chlamydia trachomatis (12). We now report on the performance of this assay for the detection of T. vaginalis in specimens from women and men.

## MATERIALS AND METHODS

This multicenter study evaluated test performance in prospectively collected first-catch urine (UR) samples from both women and men, endocervical swab (ES) specimens, clinician-collected vaginal swab (CC-VS) specimens, and patient-collected vaginal swab (PC-VS) specimens in a clinical setting. The sites participating in this study were academic medical centers, STD clinics, family planning clinics, public health departments, and clinical trial offices. The sites were located across the United States, including the West Coast, the Midwest, Southern states, and the East Coast. Subjects were eligible for enrollment regardless of the presence or absence of genital symptoms. Seventeen diverse geographic sites participated in collection of the specimens. Institutional Review Board approval was obtained for each of the collection sites.

To be eligible for study enrollment, participants needed to be sexually active, ≥14 years of age, and provide informed consent and minor assent if needed. Subjects were excluded if they had been previously enrolled in the study, had received antimicrobial therapy within 21 days prior to enrollment, had undergone a hysterectomy, or had urinated less than 1 h prior to specimen collection. Female study participants were classified as symptomatic if they reported any of the following symptoms: itching, burning, redness, soreness, or irritation of the genitals, unusual odor, abnormal vaginal discharge, coital pain, and/or dysuria. Males were classified as symptomatic if they reported any of the following signs and/or symptoms: urethral discharge, dysuria, or urethral itching or burning, burning after ejaculation, and/or dysuria. Study participants not reporting any of the above symptoms were classified as asymptomatic.

The following samples were collected from females: one PC-VS specimen (collected in a clinical setting) for testing by the Xpert TV assay, followed by two CC-VS specimens for testing by the InPouch TV culture-based method (BioMed Diagnostics, White City, OR) and the Aptima TV NAAT (Hologic, Bedford, MA) (specimens alternated), three ES specimens (the first swab for InPouch, while the second and third swabs were alternated for Aptima and Xpert assays), and 35 to 50 ml of first-catch UR for InPouch, Xpert, and Aptima assays. For males, 35 to 50 ml of first-catch UR was collected for InPouch testing, bidirectional gene sequencing of amplicons, and Xpert testing. For “collection only” participating sites, the specimens designated for Xpert testing and reference assay testing were shipped to designated testing laboratories on the same day as collection whenever possible. All Xpert testing was performed within 72 h of collection.

### Assay procedures.

Each trial specimen was collected by using the Cepheid specimen collection device (swab or urine) and tested with the Xpert TV assay. Transport reagent containing the specimen was gently inverted 3 to 4 times, followed by transferring 0.5 ml of the sample to the Xpert cartridge using the transfer pipet supplied. Aptima assay testing was performed according to the manufacturer's package insert. For InPouch testing, CC-VS and ES specimens were inoculated at the collection site. For UR cultures with the InPouch assay, 10 to 15 ml of the urine specimen was centrifuged at 500 × *g* for 5 min and the sediment inoculated into the culture pouch on-site within 1 h of collection. Culture pouches were shipped to the reference testing laboratory within 24 h of collection, with receipt of specimens occurring within 48 h of collection. These specimens were shipped at ambient temperature with two warming packs per shipping box. At the reference laboratory, cultures were incubated at 37°C and read daily on weekdays for 3 days to look for the presence of motile protozoans with characteristic morphology according to the InPouch package insert instructions.

The results of the reference tests (InPouch and Aptima for female specimens and InPouch and a validated sequencing method [bidirectional amplicon sequencing of the excess urine remaining from Xpert testing for male specimens]) were used to determine the patient infected status (PIS). The PIS was used to designate a subject as infected or not infected. The subject was considered infected if either of the reference test results were positive for T. vaginalis, while the subject was considered not infected when both reference test results were negative for T. vaginalis. Bidirectional nucleic acid sequencing was performed on specimens from women with discrepant results between Xpert testing and PIS, as well as an equal number of specimens from women who were determined to be uninfected (i.e., true negatives). For male specimens, secondary sequencing was performed on any specimens with discrepant results between Xpert testing and PIS. Quality control for the Xpert TV assay consisted of one T. vaginalis-negative and one T. vaginalis-positive external control, with both controls being run on each day that study specimens were tested. Study specimens were not run until valid test results were obtained for both the negative and positive controls. Additionally, several internal controls are built-in to the assay to monitor all aspects of the analytical process with each specimen run, including a sample processing control (SPC), a sample adequacy control (SAC), and a probe check control (PCC). The SPC is present to control for adequate processing of the target trichomonads and to monitor the presence of inhibitors in the PCR. The SAC reagents are included to detect the presence of a single-copy human gene and monitor whether the specimen contains human cells. The PCC verifies reagent rehydration, PCR tube filling in the cartridge, probe integrity, and dye stability. Only those study specimens with valid control results available for all study test methods were included in the data analyses.

Statistical analyses were performed using SAS.

## RESULTS

### Results for female subjects.

A total of 1,876 female subjects were initially enrolled in this clinical study, 1,867 (99.5%) of whom were eligible for inclusion. The nine ineligible subjects consisted of three subjects with a history of hysterectomy, two subjects with improper or incomplete informed consent, two subjects previously enrolled in the study, and two subjects who had been treated with antibiotics within the 21 days prior to study enrollment. Of the 1,867 eligible study participants, 714 (38.2%) were symptomatic and 1,153 (61.8%) were asymptomatic. The average age among eligible study participants was 33.5 years (range, 18 to 78 years).

**FIG 1:**
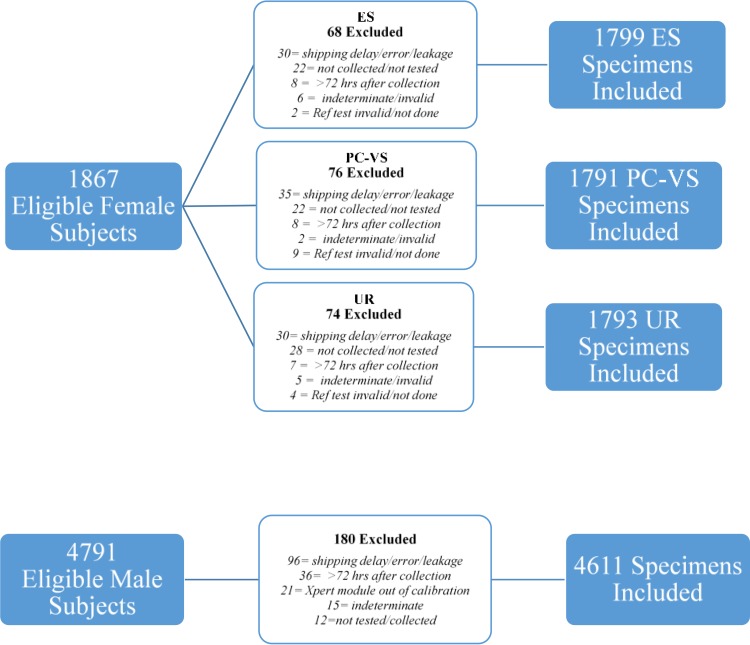
Specimen accountability for specimens from eligible female and male participants. Ref, reference.

From the 1,867 eligible female study subjects, 1,799 (96.4%) ES, 1,791 (95.9%) PC-VS, and 1,793 (96.0%) UR specimens were included in the final data set. The reasons for exclusion of each specimen type are shown in Fig. 1.

The Xpert TV (T. vaginalis) assay successfully produced results for 98.4% (5,306/5,391) of the samples from eligible female subjects on the first attempt. The 85 indeterminate results included 74 ERROR readouts (test failed, possibly because the reaction tube was filled improperly, a reagent probe integrity problem was detected, pressure limits were exceeded, or a valve positioning error was detected), 2 NO RESULT readouts (test was aborted), and 9 INVALID readouts (the SPC [sample processing control] and/or the SAC [sample adequacy control] failed—the sample was not properly processed, PCR was inhibited, or the sample was not properly collected). Eighty-three of the 85 indeterminate cases were retested; two samples were not retested. Seventy-seven of 83 indeterminate cases retested yielded valid results upon repeat assay. The overall rate of assay success was 99.9% (5,383/5,391).

Overall, relative to the patient infected status (PIS) results, the Xpert TV assay demonstrated initial sensitivity and specificity values of 98.4% and 99.7%, respectively, for urine samples, 98.9% and 98.9%, respectively, for endocervical swab specimens, and 96.4% and 99.6%, respectively, for patient-collected vaginal swab specimens (Table 1). There were no statistically significant differences in performance with respect to symptomatic status (Table 2).

**TABLE 1:** Xpert TV versus InPouch, Aptima, and PIS results for female subjects^*a*^

Sample type	Reference test or patient status	Total no. tested	Sensitivity	95% CI	Specificity	95% CI	% prevalence	PPV (%)	NPV (%)
ES	InPouch	1,799	98.7 (153/155)	95.4–99.8	97.6 (1,604/1,644)	96.7–98.3	8.6	89.3	99.9
	Aptima	1,799	100 (175/175)	98.3–99.3	98.9 (1,606/1,624)	98.3–99.3	9.7	90.7	100
	PIS	1,799	98.9 (175/177)	96.0–99.9	98.9 (1,604/1,622)	98.3–99.3	9.8	90.7	99.9
PC-VS	InPouch	1,791	96.9 (156/161)	92.9–99.0	97.7 (1,593/1,630)	96.9–98.4	9.0	80.8.	99.7
	Aptima	1,791	97.4 (186/191)	94.0–99.1	99.6 (1,593/1,600)	99.1–998	10.7	96.4	99.7
	PIS	1,791	96.4 (186/193)	92.7–98.5	99.6 (1,591/1,598)	99.1–99.8	10.8	96.4	99.6
UR	InPouch	1,793	97.7 (148/150)	95.3–99.8	97.7 (1,606/1,643)	96.9–98.4	8.4	80.0	99.9
	Aptima	1,793	99.4 (178/179)	96.9–100	99.6 (1,607/1,614)	99.1–99.8	10.0	96.2	99.9
	PIS	1,793	98.4 (180/183)	95.3–99.7	99.7 (1,605/1,610)	99.3–99.9	10.2	97.3	99.8

a Values in parentheses are the number of true positives over the total of true positives plus false negatives (sensitivity) and the number of true negatives over the total of true negatives plus false positives (specificity). PIS, patient infected status (see Materials and Methods); PPV, positive predictive value; NPV, negative predictive value; ES, endocervical swab; PC-VS, patient-collected vaginal swab; UR, urine.

**TABLE 2:** Xpert TV versus PIS results by symptomatic status for females^*a*^

Sample type	Status	No. tested	Sensitivity	95% CI	Specificity	95% CI	% prevalence	PPV (%)	NPV (%)
ES	Symp	685	100 (71/71)	94.9–100	98.5 (605/614)	97.2–99.3	10.4	88.8	100
	Asymp	1,114	98.1 (104/106)	93.4–99.8	99.1 (999/1,008)	98.3–99.6	9.5	92.0	99.8
	Overall	1,799	98.9 (175/177)	96.0–99.9	98.9 (1,604/1,622)	98.3–99.3	9.8	90.7	99.9
*P* values			0.517	−0.70, 4.48	0.331	−1.69, 0.54			
PC-VS	Symp	682	98.6 (73/74)	92.7–100	99.5 (605/608)	98.6–99.9	10.9	96.1	99.8
	Asymp	1,109	95.0 (113/119)	89.3–98.1	99.6 (986/990)	99.0–99.9	10.7	96.6	99.4
	Overall	1,791	96.4 (186/193)	92.7–98.5	99.6 (1,591/1,598)	99.1–99.8	10.8	96.4	99.6
*P* values			0.254	−1.04, 8.42	1.000	−0.77, 0.59			
UR	Symp	688	98.6 (71/72)	92.5–100	99.8 (615/616)	99.1–100	10.5	98.6	99.8
	Asymp	1,105	98.2 (109/111)	93.6–99.8	99.6 (990/994)	99.0–99.9	10.0	96.5	99.8
	Overall	1,793	98.4 (180/183)	95.3–99.7	99.7 (1,605/1,610)	99.3–99.9	10.2	97.3	99.8
*P* values			1.000	−3.25, 4.08	0.655	−0.27, 0.75			

a Values in parentheses are the number of true positives over the total of true positives plus false negatives (sensitivity) and the number of true negatives over the total of true negatives plus false positives (specificity). PIS, patient infected status; PPV, positive predictive value; NPV, negative predictive value; ES, endocervical swab; PC-VS, patient-collected vaginal swab; UR, urine; Symp, symptomatic; Asymp, asymptomatic.

Homogeneity analysis by site indicated that the results across sites were poolable for specificity. For diagnostic sensitivity, female ES specimens demonstrated nonhomogeneity; however, the overall sensitivity was high (99.5%), with all sites but one having 100% sensitivity. The one site with less than 100% sensitivity demonstrated a sensitivity of 83.3% ([10/12], 95% confidence interval [CI] 51.6% to 97.9%). Though the upper CI includes the null hypothesis, the sample size was too small for meaningful analysis. Some site-to-site variation can be expected, as well as some amount of sampling variation. Keeping this site in the pooled analysis, although it may have a slightly lower sensitivity than the other sites, was considered conservative.

### Results for male subjects.

The data in Fig. 1 illustrate specimen accountability for male subjects. A total of 4,798 male subjects were enrolled in the study, of whom 4,791 (99.9%) were eligible for evaluation. The seven ineligible subjects consisted of one subject with improper or incomplete informed consent, two subjects previously enrolled in the study, two subjects who had been treated with antibiotics within the 21 days prior to study enrollment, one subject who was not sexually active, and one subject <14 years of age. The average age among eligible male study participants with valid test results was 36.2 years (range, 16 to 78 years). Of these 4,611 study participants, 1,088 (23.6%) were symptomatic and 3,523 (76.5%) were asymptomatic. The prevalence of T. vaginalis in males across all study sites was 2.7%.

In all, a total of 4,626 (96.6%) specimens from eligible male subjects were tested using the Xpert TV assay. The urine specimens excluded consisted of 96 for shipping delays, 36 tested >72 h after collection, 21 GeneXpert modules out of calibration, and 12 not tested. Xpert TV assays for 97.7% (4,521/4,626) of the samples from eligible subjects were successful on the first attempt. The 105 indeterminate results included 84 ERROR readouts, 12 INVALID readouts, and 9 NO RESULT readouts. One hundred of the 105 (95.2%) indeterminate cases were retested; 5 samples were not retested. Ninety of 100 (90%) indeterminate cases that were retested yielded valid results upon repeat assay. Thus, the overall rate of Xpert TV assay success was 99.7% (4,611/4,626).

Overall, relative to the PIS results, the Xpert TV assay demonstrated initial diagnostic sensitivity and specificity for male urine specimens of 89.6% and 99.3%, respectively (Table 3). Secondary sequencing was performed on specimens with discordant results. The validated bidirectional sequencing procedure demonstrated that the sequencing limit of detection (LOD) was similar to the Xpert TV assay male urine analytical LOD, resulting in similar frequencies of random dropouts for specimens with low levels of T. vaginalis organisms. There were no statistically significant differences in performance with respect to symptomatic status.

**TABLE 3:** Xpert TV results versus PIS initial results based on symptomatic status for males^*a*^

Status	No. of UR samples tested	Sensitivity	95% CI	Specificity	95% CI	% prevalence	PPV (%)	NPV (%)
Symp	1,088	87.5 (28/32)	71.9–95.0	99.8 (1,054/1,056)	99.3–99.9	2.9	93.3	99.6
Asymp	3,523	90.3 (84/93)	82.6–94.8	99.2 (3,401/3,430)	98.8–99.4	2.6	74.3	99.7
Total	4,611	89.6 (112/125)^*b*^	83.0–93.8	99.3 (4,455/4,486)^*c*^	99.0–99.5	2.7	78.3	99.7
*P* values		0.738	−15.8, 10.1	0.020	0.25, 1.06			

a Values in parentheses are the number of true positives over the total of true positives plus false negatives (sensitivity) and the number of true negatives over the total of true negatives plus false positives (specificity). PIS, patient infected status; UR, urine; PPV, positive predictive value; NPV, negative predictive value; Symp, symptomatic; Asymp, asymptomatic.

b Results from secondary sequencing: 9 of 13 false negatives were T. vaginalis negative and 4 of 13 were T. vaginalis positive.

c Results from secondary sequencing: 27 of 31 false positives were T. vaginalis positive and 4 of 31 were T. vaginalis negative.

## DISCUSSION

Trichomoniasis is a highly prevalent STD and presents with a spectrum of symptoms or with no symptoms (3). In women, the prevalence of infection spans the reproductive years and beyond, with some studies showing high rates in older women (13). The epidemiology is less well understood in men, although a recent study showed that age over 40 years was also associated with T. vaginalis-associated sexually transmitted infections (STIs) (14). Accurate identification of trichomoniasis is important for several reasons, including optimizing treatment, realizing the need for treatment of the sexual partner(s) (15), and the potential for prevention of associated public health consequences, such as preterm birth and the acquisition and transmission of HIV (6–8). The CDC has recently recommended NAAT as the preferred diagnostic modality in light of their superior sensitivity compared to those of direct microscopy or culture-based methods for detecting T. vaginalis (2). Diagnostic testing is recommended for symptomatic women, and screening should be considered for individuals with multiple sex partners, persons who exchange sex for payment, use drugs, and/or have a history of STDs, and women in high-prevalence settings, such as STD clinics and correctional facilities (2). Annual screening is recommended for women with HIV infection due to the high rates of T. vaginalis infection in this population (16). Rescreening is recommended within 3 months of the initial diagnosis due to high recurrence rates (17). Currently, there are no firm screening recommendations for men due to the fact that an FDA-cleared test has only recently become available. However, men attending STD clinics should be considered for screening (2). A recent study showed a prevalence rate of nearly 10% among men attending an STD clinic in Birmingham, AL (14).

Although other NAAT in addition to the Xpert TV assay have also recently been FDA cleared for the diagnosis of T. vaginalis in women (11, 18), the Xpert TV assay can provide on-demand results in 63 min or less, with early termination for positive results within 40 min. This quicker turnaround time makes the GeneXpert platform ideal for use in high-risk settings where diagnosis and treatment could ideally take place in real time for optimal public health control of this STD.

The platform is easy to use and, thus, suitable for in-clinic testing at the point of care (POC). Furthermore, the equally high diagnostic sensitivities and specificities demonstrated here for urine, self-collected vaginal swabs, and endocervical swabs in symptomatic and asymptomatic patients make the assay an important diagnostic screening tool for patients in high-risk settings like STI clinics and emergency departments, as well as gynecology clinics with laboratory facilities. Of note, the majority of men were asymptomatic, suggesting that screening for trichomoniasis should be considered for high-risk men, such as those attending STD clinics.

The availability of NAAT testing for T. vaginalis in men is a welcome addition from a public health perspective and has been a key element missing for the control of this STD. The sensitivity and specificity of the Xpert TV assay relative to the PIS results were both high, 97.2% and 99.9%, respectively. However, it should be noted that InPouch cultures for this study followed the current manufacturer's package insert at the time of the study (Biomed Diagnostics document no. 100-001, revision K), which instructed users to incubate and read cultures for up to 3 days. A study conducted by Rivers and colleagues (19) showed that an additional 17.2% of cultures were identified as positive when cultures were incubated and read up for to 5 days. Thus, a study comparing the Xpert TV assay directly to InPouch cultures incubated for 5 days would need to be conducted in order to determine whether the sensitivity of the Xpert assay would be slightly lower.

In summary, T. vaginalis is a highly prevalent STI associated with increased risk of HIV acquisition and transmission, as well as preterm births. The availability of an FDA-cleared test for men is especially welcome. Recent advances in molecular diagnostics for this highly prevalent infection, if implemented, should begin to reduce the burden of disease.
